# Determinants to late antenatal clinic start among pregnant women: the case of Saint Elizabeth General Hospital, Shisong, Cameroon

**DOI:** 10.11604/pamj.2020.35.112.18712

**Published:** 2020-04-10

**Authors:** Mbinkar Adeline Venyuy, Samuel Nambile Cumber, Claude Ngwayu Nkfusai, Fala Bede, Yunga Patience Ijang, Emerson Wepngong, Solange Ngo Bama, Joyce Mahlako Tsoka-Gwegweni, Pierre Marie Tebeu

**Affiliations:** 1 Department of Public Health, School of Health Sciences, Catholic University of Central Africa, Box 1110, Yaoundé, Cameroon; 2 Cameroon Baptist Convention Health Services (CBCHS), Yaoundé, Cameroon; 3 Centre for Health Systems Research & Development, University of the Free State, Bloemfontein, South Africa; 4 Faculty of Health Sciences, University of the Free State, Bloemfontein, South Africa; 5 School of Health Systems and Public Health, Faculty of Health Sciences, University of Pretoria, Pretoria, South Africa

**Keywords:** Maternal health, child health, pregnancy, health system, antenatal clinic, pregnant women, health institutions, Cameroon

## Abstract

**Introduction:**

To improve maternal health, barriers that limit access to quality maternal health services must be identified and addressed at all levels of the health system. The World Health Organisation (WHO) cites distance to health facility and inadequate health institutions as factors that prevent women from receiving or seeking care during pregnancy and childbirth. Specifically, we intended to determine factors associated with late start of late Antenatal Care (ANC) among pregnant women in the Saint Elizabeth General Hospital Shisong (SEGHS), Cameroon.

**Methods:**

This was a cross sectional study carried out from the 24^th^ October to 24^th^ November 2016. A total of 602 pregnant women were recruited from ANC units of SEGHS and its satellite institutions. The outcome variable was gestational age at start of ANC (estimated by counting from last menstrual period to day of first ANC consultation) while the independent variables were individual, community and institutional factors. Data was analyzed using Epi info version 7. Chi square test was used to appreciate the influence of different variables on risk of late ANC initiation (> 14 weeks of pregnancy). The level of significance was set out at (p: < 0.05).

**Results:**

Out of the 602 pregnant women included in our study, 75% initiated ANC late (after 14 weeks of pregnancy). Factors associated with late ANC start were; age (p = 0.001), level of education (p = 0.002), marital status (p = 0.016), religion (p = 0.034), parity (p = 0.001), having a source of income (p=0.001), cost of services (p = 0.010), distance to health facility (p = 0.021) and dissatisfaction with previous ANC services (p = 0.014).

**Conclusion:**

Cameroon is one of the countries with a high maternal mortality ratio. WHO estimated it to be 529 per 100000 live births in 2017. Prompt and adequate ANC services can improve on maternal and child outcomes of pregnancy. The results of this study suggest tackling issues related to cost of ANC services and improving geographical (distance) barrier to accessing ANC services (in addition to addressing other identified measures) may lead to an increase in pregnant women starting ANC early and thus potentially improve pregnancy outcomes.

## Introduction

Accessibility related factors influencing late ANC start include: long distance from facilities providing services, mode of transport, working hours, booking appointments, and direct or indirect discrimination by prenatal care providers. The results of a review study showed that availability of prenatal care services is related to the use of these services [1]. To improve maternal health, barriers that limit access to quality maternal health services must be identified and addressed at all levels of the health system. WHO cites distance to health institution and inadequate health institutions in her fact sheet as factor that prevent women from receiving or seeking care during pregnancy and childbirth [2]. In the Copper Belt Province of Zambia, rural pregnant women were 3.4 times (adjusted OR 3.4, 95% Confidence Interval (95% CI): 1.18-9.70) more likely to initiate ANC late because of long distance to health facilities which acted as a discouragement factor [3]. Christian Banda in her thesis carried out in the Ntchisi District in Malawi, was able to demonstrate that long distance to health facilities was associated to the low use of antenatal services by pregnant women [4].

African Progress Penal Policy (APPP (2010)) [5] puts stress on poor road infrastructure and transportation which present another hurdle to effective care. Especially in rural areas, clinics are often too far away or otherwise inaccessible. Frequently there are no roads to the nearest health facility, or existing roads are impassable due to road quality, terrain, natural disasters or the rainy season. A study carried out in Cameroon [6] identified distance to health facility as a risk factor for poor pregnancy outcomes in adolescents. Late initiation and inadequate use of prenatal care services are independently associated with multiple variables, including demographic characteristics, socio-economic factors, predisposing cultural and religious factors, social support, factors related to healthcare providers, women's awareness and attitude, unintended pregnancy, high-risk medical or obstetric history, and health behaviours [7]. Therefore, we intended to identify and determine factors associated with late prenatal care initiation among pregnant women in the Saint Elizabeth General Hospital Shisong Cameroon and its health institutions.

## Methods

**Study design:** the study was a quantitative research. Cross-sectional analytic study was done using clinical method.

**Duration of study:** the period of study involved then period during which data was collected, it ran for a month (24^th^ October to 24^th^ November 2016).

**Study population:** the target population was all pregnant women of child bearing age from 15 years and above attending ANC in the Saint Elizabeth Catholic Hospital and its health institutions.

**Inclusion criteria:** all women of child bearing age from 15 years and above who gave consent (those of 18 years and above) or for minors (18 years below), whose parents gave their consent; All pregnant women who had started antenatal consultation and who accepted to participate in our study.

**Exclusion criteria:** all pregnant women who were seriously sick; all pregnant women who started their ANC in another health institution different from these two; all women of child bearing age below the age of 18 years whom parental consent was not granted.

**Sampling methods:** the sampling method employed was non-probabilistc convenience sampling. The calculation of the sample size was done using the formula below using a prevalence of 50% in order to obtain the maximum sample size possible. Based on this, we determined our minimum sample size to be 381 and taking into account for a non-response rate of 10% the final calculated sample size was 420. However, in our study, 602 pregnant women were taking part.

$$n=\left[p\;*\;(1-p)*z^{2}{}_{\left(1-\frac{\alpha }{2}\right)}\right]/d^{2}$$

PΟ = Proportion of women starting ANC late (P = 50%); a = 0. 05→Za = 1.95; d is the margin of error (d = 5%).

**Study variables and operational definitions:** for the purposes of this study, the independent variables were grouped into individual, community (social) and health systems related factors. Individual factors were: age, level of education, marital status, religion and parity. Social factors included: who was responsible for decisions, whether the spouse was employed or not, having a source of revenue, and whether there was peer influence. The health system related factors were: opinion about quality of services, cost of prenatal services, distance from health facility and satisfaction with previous ANC services. The dependent variable in our study was gestational age of pregnancy (calculated from LMP) at the time of initiating ANC. It was categorized as late if more than 14 weeks or not late if it was 14 weeks or less.

**Study procedure of data collection:** this was a hospital-based survey where all participants who consented were interviewed using a structured questionnaire filled in the hospital. Prior to use in the study participants, a total of 20 questionnaires were pretested at the Catholic University of Central Africa among female students aged 15 years and above with the aim of revising poorly structured questions, estimate the average time required to fill the questionnaire and thus validate the use of the questionnaire in our context. It was estimated that; each questionnaire could be administered for 30-45 minutes after the pretest. A total of 602 questionnaires were administered to women greater than or equal to 15 years of age attending ANC in the Saint Elizabeth Catholic Hospital and its health institutions with objectives intended to identify and determine the determinants of late ANC initiation among pregnant women in the Saint Elizabeth General Hospital Shisong and its Health Institution, Cameroon. The questionnaire which was sub-divided into two parts was used for data collection. It consisted: (1) Socio-demographic characteristics of the pregnant women attending ANC unit of the Saint Elizabeth general hospital and its health institutions; (2) Health system determinants are related to late ANC attendance among women in the ANC unit in the Saint Elizabeth general hospital and its health institutions.

**Data analysis:** data was entered using Microsoft Excel and analyzed using Epi Info version 7.0. Frequencies and percentages were determined for categorical variables. Means and standard deviations (mean ± SD) were calculated for continuous variables. To investigate associations between the independent variables and the outcome variable we used Chi-Square test as they were categorical variables. Fischer's exact test was used in cases were conditions for Chi-Square test were not met. Statistical significance was set at p < 0.05.

**Ethical consideration:** ethical approval for the study was obtained from the Institutional Research Ethics Committee for Human Health (CIERSH) at the School of Health Sciences of the Catholic University of Central Africa. Administrative clearance was obtained from the Director of the saint Elizabeth Catholic General Hospital were our research took place. In this study the confidentiality of patient information and identity was respected. Written consent was obtained from all participants and confidentiality of participants was ensured by using anonymous questionnaires. Participation in the study was without any coercion and participants were free to withdraw or ask their information withdrawn at any time during the study.

## Results

### Distribution of pregnant women according to the age of initiation of ANC

From our dependent variable, we realized that 75% of women-initiated ANC late (after 14 weeks of gestation) compared to 25% of those who initiated earlier before 14 weeks of gestation. This also implies that, 454 women-initiated ANC later against 148 women who initiated earlier.

### Individual determinants of late ANC among women in the SEGHS and its health institutions

Late ANC initiation is mostly observed from the age range of 30-44 years, (53.30% Vs 32.43%). Women with low level of education (50.11% Vs 36.05%). The same phenomenon is observed in unmarried women (87.37% Vs 47.57%). Christians too were reported to start ANC late (85.81% Vs 77.75%). A majority of the women have had 2-9 pregnancies (74.67% Vs 55.41%) and women who had 2-8 children (55.95% Vs 68.49%). Late ANC initiation is more illustrated in women who do not have a permanent source of income (72.03% Vs 32.43%) (Table 1).

**Table 1:** Individual characteristics of the respondents according to ANC initiation

Characteristics	Age of initiation of ANC			p-value
	>14 weeks N = 454 n(%)	≤14 weeks N = 148 n(%)	Total 602 n(%)	
**Age**				0.001*
30-44	242(53.30)	48(32.43)	290(48.17)	
15-29	212(46.70)	100(67.57)	312(51.83)	
**Level of education**				0.002*
≤Primary	228 (50.11)	53(36.05)	281(46.68)	
≥Secondary	227(49.89)	94(63.95)	321(53.32)	
**Marital status**				0.016*
Not married	346(87.37)	98(47.57)	444(73.75)	
Married	50(12.63)	108(52.43)	158(26.25)	
**Religion**				0.034*
Christians	127(85.81)	353(77.75)	480(79.73)	
Muslims	21(14.19)	101(22.25)	122(20.27)	
**Gravidity**				0.001*
2-9	339(74.67)	82(55.41)	421(69.93)	
1	115(25.33)	66(44.59)	181(30.07)	
**Parity**				0.001*
4-8	254(55.95)	128(86.49)	382(36.54)	
0-3	200 (44.05)	20(13.51)	220(63.46)	

### Community determinants of late ANC among pregnant women in the SEGHS and its health institution

It is known that the environment of an individual influence's certain decisions in life. This could be applied also to the choice of when to begin ANC among the women of this community. It is important to know those community factors that can influence the time of ANC initiation (Table 2).

**Table 2:** Community characteristics with respect to ANC initiation

Characteristics	Age of initiation of ANC			p-value
	>14 weeks N = 148 n(%)	≤14 weeks N = 454 n(%)	Total 602 n(%)	
Decision-maker				
**Couple**	383 (84.36)	117(32.40)	500(83.06)	1.135
**Others**	71(15.64)	31(20.95)	102(16.94)	
Financial supporter				
**Others**	85 (25.99)	28 (28.00)	113(26.46)	0.690
**Parent of child**	242(74.01)	72 (72.00)	314(73.54)	
Husband’s profession				
**Non C S**	53 (15.36)	11 (11.22)	64(14.45)	0.304
**CS**	292 (84.64)	87 (88.78)	379(85.55)	
Transport fare				
**0-500**	314 (69.16)	101 (64.24)	415(68.94)	0.834
**600 and above**	140 (30.84)	47 (31.76)	187(31.06)	
Source of income				
**No**	327 (72.03)	48(32.43)	375(62.29)	0.001*
**Yes**	127(27.97)	100(67.57)	227(37.71)	

**others**: other family members

* **CS:** civil servants

### Health system related characteristics to late ANC among pregnant women in the SEGHS and its health institution

These are the factors related to the health system of these health institutions that can have an impact on the timing of ANC by the women who use the services. *Influence of distance on decision to start ANC on time: this is to understand whether the fact that the women stay far can cause her to book late for ANC. Those who said cost of ANC was not affordable were (58.15% Vs 49.95%). The same phenomenon was observed to those who considered not being influenced by other people's opinion (74.3% Vs 37.89%). Women who said distance did not hinder their decision to start ANC were (78.9% Vs 21.05%) and those who were not satisfied with care offered were (52.20 Vs 40.54%). There were homogenous factors like; quality of care offered, cost of ANC and distance in KM to health institutions (Table 3).

**Table 3:** Health system determinants with respect to age of ANC initiation

Characteristics	Age of initiation of ANC		Total	p-value
	>14 weeks N = 454 n(%)	≤14 weeks N = 148 n(%)	Total 602 n(%)	
**Quality of care offered**				
Very Good	351 (77.31)	107 (72.30)	458(76.08)	0.214
Good	103 (22.69)	41(27.70)	144(23.92)	
**Cost Affordability**				
Not affordable	264 (58.15)	68 (49.95)	332(55.15)	0.010*
Affordable	190 (41.85)	80(54.05)	270(44.85)	
**Cost of ANC**				
400 -15000	150(33.04)	52 (35.14)	202(33.55)	0.639
100 -3900	304(66.96)	96 (64.86)	400(66.45)	
**Opinion of others**				
No	110(74.32)	172(37.89)	282(46.84)	0.001*
Yes	38 (25.68)	282 (62.11)	320(53.16)	
**Distance**				
≤5km	364 (80.18)	116 (78.38)	480	0.637
>5km	90 (19.82)	32 (20.27)	122	
**Influence of distance**				
Yes	270 (59.47)	72 (48.65)	342(56.81)	0.021*
No	184 (40.53)	76 (51.35)	260(43.19)	
**Satisfaction at ANC**				
Not satisfied	237(52.20)	60(40.54)	297(49.34)	0.014*
			

## Discussion

In our results, women aged 28 years and above showed little interest in the use of ANC services during the first trimester than younger women [8]. In northern Ethopia, young pregnant women (aged 25 and below) were found to be more likely to commence ANC within the recommended time compared to their older counterparts Results compared to ours could be justified by the fact that all the studies were carried out in rural areas. The social environment greatly influences behavior. Ajzen *et al.* makes us understand that one could choose how to respond to a given situation based on the consequential outcome expected. A great solidarity exists among different health systems that influence behaviors and attitudes towards attending first ANC. The health behavior model could also be used to explain this. If the barriers to early and prompt ANC initiation are great in a particular group of persons in a particular environment, they would never perceive benefits that they could have initiating ANC during the first trimester. The different health institutions should take into consideration cultural diversities and beliefs to sensitize need for first ANC. Parity in our study was associated with early or late start of ANC. Primiparous women started ANC late compared to multiparous women. (p: 0.035). This could be because of positive past antenatal care experience(s) before current pregnancy. It could also be as a result of the fact that these women are living in poverty with low family income in conformity with this study, a study in Rwanda proved parity to be associated to timing of ANC initiation [9]. Women or respondents who reported that the cost of ANC was unaffordable were more likely to initiate ANC late compared to their counterparts (p =0.010). This could be as a reason of good management when they become aware of their pregnancy or better still it could be for their pregnancies are wanted pregnancies. A study in Nigeria by [10] reported that most women initiate ANC late because of lack of finances for the use of ANC services. The financial barrier as an influential factor to late ANC is seen in studies of [11] in Tanzania, [12] in Muea, Cameroon, [13] in a study carried out in Guinea, shows that most women had difficulties initiating ANC during the first trimester of pregnancy because it is not always easy to afford for the money to pay ANC fee with the first visits being sometimes excessively expensive. To these women and even women who had not attended ANC before when interviewed said this barrier of cost of services would not be difficult to overcome. The behavior of pregnant women with respect to late ANC could be linked to the lack of finances where the first visit often demands more. These women express that it is not always easy to afford the resources needed given the standard of life in rural areas. With the unavailability of finances, these women find it difficult to attend the ANC early as they may not be able to afford the services. When there is this financial barrier the ease to care is obstructed thus delay in ANC attendance. Finlayson & Downe (2013) reveals that the costs fee for ANC which is high and most often unaffordable, both direct and indirect) of visiting antenatal facilities were viewed as a significant factor in restricting or inhibiting access to antenatal care [14]. There is a need for partners and families to support these women financially towards positive pregnancy and child birth outcomes.

Distance to the health center was significantly associated with late ANC initiation in our study. More pregnant women who lived far from nearest health facilities (more than 5km) started ANC late (after the 14^th^ week of pregnancy) compared to those who were estimated to live at less than 5Km from the health facility (p=0.021). A study carried out in Rwanda by [9] feeling that distance to health facility is a problem that had an influence of decision taking on ANC initiation (OR: 1.20, 95% CI: 1.04-1.38). A study by [1] contradicts our study by reporting a significant association between the initiation of prenatal care and distance from facilities providing these services. Lack of access to services due to long distance and transport-associated problems are among the main reasons for not receiving prenatal care on time in most rural areas. Moreover, most studies report that distance is a hindering factor to early initiation of ANC. Banda *et al.* (2012) [3] in Zambia showed pregnant women in rural areas were 3.4 times (AOR 3.38, 95% CI 1.18-9.66) more likely to initiate ANC late because of long distance to health facilities which acted as a discouragement factor. Banda (2013) [4] in the Ntchisi District in Malawi, also showed that long distance to health facilities was associated to the low use and untimely use of antenatal services by pregnant women as well as influenced the number of ANC visits. This brings about the need for PEER educators to be used to follow up women in their various health sectors during campaign programs. Satisfaction with previous ANC services was reported to by slightly more than half of the respondents. This was lower than the 81.1% in Ibadan, Nigeria by [15]. The satisfaction of ANC influences the initiation of ANC. Women reported that they could not go to the hospital for ANC initiation if they did not have money. This emphasis on the importance of meeting individual needs through adequate and sufficient personnel. Due to the fact that the personnel are limited, work load become a barrier to meeting the needs of these women reason why the hospital has to employ more persons in this sector. Women who reported that other women did not see quality of care as being satisfying had a 4.75 risk of initiate ANC late than their counterparts. The results showed 297/602 (49.34%) of women were not satisfied on the care they received. This was related among others to long waiting hours (58.44%), numerous visits (21.81%), and attitude of workers (15.44%). This actually impacted on their attending and initiating ANC on time. The fact that more of them perceived the care as being satisfactory could be because of their low expectations of health care services. For during the investigators collection period where she had to recruit women during their ANC visits, it was observed that the quality of care was low. Their answers could thus have been influenced by the fact that they were interviewed at the health facility. This is similar to what was reported by [11] in Tanzania. Also, it could be related to the fact that these women had limited knowledge on the ANC package or what to expect. For as reported by in South Sudan, that these women either started their consultation in the second, third or fourth trimester or never attended at all. There is the need for good health education with modern tools to be put in place to help counsel the negative reasoning women bring from quarters that hinder them to apply what they are told during health education. This is to help reduce maternal and infant mortality rate as a whole.

### Study limitations

This study has some limitations. One of the limitations is the fact that pregnant women who attend antenatal care in other private and public health facilities are not included in the study. Moreover, gestational age was determined based on women's reports of their Last Menstrual Period (LMP). Ultrasound scan to confirm gestational age was not performed; hence, this may have caused inaccuracies in measurement of gestational age. Moreover, this is a cross-sectional analytic study whose findings are not generalized to a general population because study was carried out in a particular district area. As a cross-sectional study, the associations observed between the explanatory variables and the outcome does not show causal relationship.

## Conclusion

Reproductive Health is a concern of any age through the life cycle and represents more than 80% of health issues. The forecasts on the reproductive health services must take into account the population’s structure, its age, its diversity and its dynamics for specific needs within the various groups. We had as objectives identifying individual factors which among that proved significant were, the age of woman, marital status and gravidity. As to community factors, permanent source of income revealed itself as the center concerned and health institutional factors that will influence late ANC were cost affordability, cost price of ANC, opinion of others on when to initiate, influence of distance and satisfaction derived from ANC. Findings of our study revealed that 75% of the pregnant women who participated booked for ANC only after 14 weeks of gestational age. Late ANC attendance was linked to factors that ranged from individual, community and health institutions as earlier explained. This study provides necessary information to contribute to the reduction of late attendance of ANC thus providing chances for oriented interventions towards the detection of maternal problems which could be fatal to both her health and that of her baby to be.

### What is known about this topic

- Antenatal care visit attendance, intermittent preventive treatment during pregnancy (IPTp) and malaria parasitaemia at delivery;
- Birth preparedness and complication readiness practice and associated factors among pregnant women;
- Perceptions of antenatal care services by pregnant women attending government health centres in the Buea Health District, Cameroon: a cross sectional study.

### What this study adds

- Findings of our study revealed that 75% of the pregnant women who participated booked for ANC only after 14 weeks of gestational age;
- Late ANC attendance was linked to factors that ranged from individual, community and health institutions as earlier explained;
- Evaluating different groups and diverse cultural aspects brings out the understanding of how different groups behave with regards to ANC 1 attendance.

## Competing interests

The authors declare no competing interests.
